# Whole-Genome Sequence of a Human Monkeypox Virus Strain Detected in Egypt

**DOI:** 10.1128/mra.00006-23

**Published:** 2023-05-08

**Authors:** Wael H. Roshdy, Rabeh El-Shesheny, Yassmin Moatasim, Mina N. Kamel, Shaymaa Showky, Mokhtar Gomaa, Amel Naguib, Nancy El Guindy, Manal Fahim, Mohamed Khalifa, Ramy Galal, Mohamed Hassany, Ahmed Kandeil, Mohamed A. Ali, Amr Kandeel

**Affiliations:** a Central Public Health Laboratory, Ministry of Health and Population, Cairo, Egypt; b Centre of Scientific Excellence for Influenza Viruses, National Research Centre, Giza, Egypt; c Department of Epidemiology and Surveillance, Preventive Sector, Ministry of Health and Population, Cairo, Egypt; d Public Health Initiative, Cairo, Egypt; e National Hepatology and Tropical Medicine Research Institute, Ministry of Health and Population, Cairo, Egypt; f Preventive Sector, Ministry of Health and Population, Cairo, Egypt; DOE Joint Genome Institute

## Abstract

Monkeypox virus has recently been detected in multiple countries. Two cases of monkeypox virus were reported in Egypt as part of an ongoing international outbreak. We report the whole-genome sequence of a monkeypox virus that was retrieved from the first confirmed case in Egypt. The virus was fully sequenced on the Illumina platform, and phylogenetic analysis demonstrated that the current monkeypox strain is closely related to clade IIb, which caused recent multicountry outbreaks.

## ANNOUNCEMENT

Monkeypox (Mpox) virus is a member of the *Orthopoxvirus* genus of the *Poxviridae* family of enveloped viruses, harboring a linear double-stranded DNA genome of around 200 kb. Mpox virus is an emerging zoonotic pathogen that was first isolated from monkey in 1958. Mpox symptoms are similar to those of smallpox infection, including fever, headache, muscle ache, lymphadenopathy, and a characteristic rash that develops into papules, vesicles, and pustules, which eventually scab over and heal (1, 2).

As of 10 October 2022, 71,237 laboratory-confirmed cases and 1,097 probable cases, including 26 deaths, had been reported to the World Health Organization (WHO) from 107 countries. According to the WHO nomenclature, Mpox is genetically divided into two clades; clade 1 Mpox virus, formerly known as the Congo Basin (Central African) clade, is associated with high mortality rates, and clade 2 Mpox virus, formerly known as the West African clade, is associated with milder infection and lower mortality rates (3, 4). On 8 September 2022, the Egyptian Ministry of Health announced that it had detected a first Mpox case in Egypt. The case involved a 42-year-old man with residency in a European country. The patient was isolated in a hospital, and his condition was stable. A second case was detected on 26 September 2022. Both patients were examined at Abbassia Fever Hospital (Cairo, Egypt) and diagnosed as suspected cases of Mpox virus infection. Pustule, nasal, and oropharyngeal swab samples were collected in viral transport medium for delivery to the Central Public Health Laboratory (CPHL) for testing with Mpox virus real-time PCR assays, i.e., the LightMix modular orthopoxvirus assay (research use only [RUO]) (TibMol-biol, Berlin, Germany) and LightMix modular monkeypox virus assay (RUO) (TibMol-biol), and positive results were confirmed using the generic G2RG_G primers (5).

Viral DNA was extracted using a QIAmp DNA minikit (Qiagen, Germany). To amplify the entire Mpox virus genome, we used two pools of the primalSeq v2 primer sets (6). Multiplex PCR was performed using Phusion high-fidelity DNA polymerase (Thermo Fisher Scientific). The PCR amplicons of pool 1 and pool 2 reactions for the clinical sample from the second case were combined. DNA libraries were prepared using CovidSeq kits (Illumina, San Diego, CA, USA) according to the manufacturer’s instructions. Pooled libraries were sequenced with an Illumina MiSeq personal genome sequencer with 150-bp paired-end reads. Default parameters were used for all software unless otherwise specified. A total of 19,679,577 reads were used for assembly based on a reference genome sequence (GenBank accession number MT903345.1) through the tools Merge overlapping pairs, Trim reads, Map reads to reference, and Extract consensus sequence using CLC Genomics Workbench v21 (CLC Bio, Qiagen).

The complete genome of the MOH-NRC-0002/2022 strain consisted of 197,201 bp (G+C content, 33.01%), with 192.1× coverage, and has been submitted to GenBank under accession number OP597769. To determine the viral lineages, we used Nextclade v2.9.1 (https://clades.nextstrain.org). Results showed that the Mpox strain MOH-NRC-0002/2022 belongs to lineage A.2.1 in clade IIb. The full genome sequence of the MOH-NRC-0002/2022 strain was compared to all other complete Mpox virus sequences available in the NCBI database on 15 January 2023 using BLAST (7). The analysis revealed that the Egyptian strain shared 99.97% and 99.94% nucleotide identity with the closest strains MPXV-UK_P3 (GenBank accession number MT903345.1) and MPXV_USA_2021_TX (GenBank accession number ON676707.1), respectively. Gigante et al. showed that lineage A.2 had several mutations, compared to the dominant lineage B.1, in 2022 (8). Mpox virus lineage A.2 strains have been detected in the United States, Thailand, the United Kingdom, Portugal, and Slovenia and are common in India. In summary, we report the complete genome sequence of Mpox virus strain MOH-NRC-0002/2022, which was isolated from an Mpox patient in Egypt. Rapid genetic characterization of newly emerging viruses is a fundamental tool for understanding virus dynamics. Such knowledge is vital to predict and fight potential pandemics that could threaten public health.

This work was approved by the Medical Research Ethics Committee of the National Research Centre (Egypt) (protocol number 1-4-6).

### Data availability.

The genome sequence of the Egyptian Mpox virus strain was submitted to GenBank and is available under accession number OP597769.1. The accession numbers for the Illumina MiSeq sequence raw reads in the NCBI Sequence Read Archive (SRA) are PRJNA887588 (BioProject), SAMN31178476 (BioSample), and SRR21841757 (SRA) (hMpxV/Egypt/MOH-NRC-0002/2022).
